# Efficacy of Graphene-Based Nanocomposite Gels as a Promising Wound Healing Biomaterial

**DOI:** 10.3390/gels9010022

**Published:** 2022-12-28

**Authors:** Dilip Kumar Shanmugam, Yasasve Madhavan, Aashabharathi Manimaran, Gobi Saravanan Kaliaraj, Karthik Ganesh Mohanraj, Narthana Kandhasamy, Kamalan Kirubaharan Amirtharaj Mosas

**Affiliations:** 1 Centre for Nanoscience and Nanotechnology, Sathyabama Institute of Science and Technology, Chennai 600119, India; 2 Department of Oral Medicine and Radiology, Faculty of Dental Sciences, Sri Ramachandra Institute of Higher Education and Research, Chennai 600116, India; 3 Department of Biotechnology, Sree Sastha Institute of Engineering and Technology, Chembarambakkam, Chennai 600123, India; 4 Department of Anatomy, Saveetha Dental College and Hospital, Chennai 600077, India; 5 Coating Department, FunGlass—Centre for Functional and Surface Functionalized Glass, Alexander Dubcek University of Trencin, 91150 Trencin, Slovakia

**Keywords:** microbial infection, aloe vera, hydrogel, graphene oxide (GO), reduced graphene oxide (rGO), wound healing

## Abstract

The development of biocompatible nanocomposite hydrogels with effective wound healing/microbicidal properties is needed to bring out their distinguished characteristics in clinical applications. The positive interaction between graphene oxide/reduced graphene oxide (GO/rGO) and hydrogels and aloe vera gel represents a strong strategy for the advancement of therapeutic approaches for wound healing. In this study, the synthesis, characterization, and angiogenic properties of graphene-based nanocomposite gels have been corroborated and substantiated through several in vitro and in vivo assays. In this respect, graphene oxide was synthesized by incorporating a modified Hummer’s method and ascertained by Raman spectroscopy. The obtained GO and rGO were uniformly dispersed into the aloe vera gel and hydrogel, respectively, as wound healing materials. These formulations were characterized via in vitro bio-chemical techniques and were found suitable for the appropriate cell viability, attachment, and proliferation. In addition, in vivo experiments were conducted using male Wistar rats. This revealed that the GO/rGO-based gels stimulated wound contraction and re-epithelialization compared to that of the non-treatment group. From the study, it is suggested that GO/rGO-based aloe vera gel can be recommended as a promising candidate for wound healing applications.

## 1. Introduction

Wound healing is a convoluted progression that integrates a variety of tissue and cell types and majorly includes hemostasis, proliferation, inflammation, and remodeling [1,2]. One of the numerous threats associated with wound healing is the prevalence of contamination of wound surfaces by antibiotic-resistant bacteria [3,4]. The emanation is quite drastic, which could lead to a protracted bacterial infection that seriously obstructs the healing process [5]. Such bacterial defilement is typically examined by engaging surgical techniques and/or the application of antibiotic medications [6]. Nonetheless, the complication of tissue resection, along with the ascent in antimicrobial resistance, hinders the overall therapeutic outcome. This situation highlights the significance of recognizing state-of-the-art substituting antimicrobial agents that can resolve the clinical challenge posed by infectious wounds.

Researchers and the clinical market are interested in hydrogel-based wound dressing among various wound dressing techniques [7]. In general, polymeric substances called hydrogels are hydrophilic and adapt to absorbing water [8]. Infected wound dressings made of hydrogel keep the environment moist and give the wound’s surface sufficient air conditioning. In the meantime, they do not immediately adhere to the wound’s surface and reduce pain, particularly when the dressing is removed. In swollen conditions, hydrogels mimic live tissues, showing their rubbery and squishy nature. Additionally, it might accelerate autolytic debridement and advance re-epithelialization, making hydrogel-based wound dressings the best dressing material [9].

In addition, hydrogel preparations in the pharmaceutical industry include a significant amount of water and serve to reduce pain when applied, especially to mucous membranes and burned or wounded skin which makes them preferable to creams [10]. When used for dermatological purposes, hydrogels have a number of advantages over ointments, such as being emollient, greaseless, and thixotropic. Hydrogels spread more readily are simple to remove, do not leave stains, work well with a variety of excipients, and are water-soluble or miscible [11]. As a result, creating hydrogel-based wound dressings employing a variety of biocompatible matrices and physiologically active materials such as alginate, polyacrylic acid (PAA) (carbopol), chitin, and chitosan became of great interest. As a smart gel or environmentally friendly polymer, carbopol 934 is also well known [12]. Different carbopol polymers are essential in the creation of stimuli-responsive hydrogels because they alter their swelling behavior in response to environmental factors including pH, light, an electric field, or temperature [13]. 

Later, researchers used natural substances like therapeutic plant components, which were integrated into the wound formulations to boost bioactivity, in addition to hydrogels [7,8]. Among the various plant therapeutic agents, aloe vera plants exhibit unique characteristics that include enzymes, carbohydrates, minerals, and vitamins and have excellent antioxidant, anti-bacterial, and antiviral properties [14]. The glucomannan compound found in aloe vera plants promotes the growth of several cell types, improve anti-inflammatory property, influence fibroblast growth factor release, cell proliferation, and collagen synthesis [15,16]. Additionally, it promotes the healing process by triggering fibroblast proliferation, angiogenesis, and the synthesis of the extracellular matrix (ECM) [16].

The development of chemical and biological sensors, catalysts, cancer medication delivery [17], nanomedicine, imaging systems, and antibacterial agents all make use of the unique fundamental features of 2D structures of GO and rGO [18]. With hydroxyl, epoxide, and carboxyl groups on its exterior, GO/rGO is an oxidized carbon layer that improves biological interactions and scaffold hydrophilicity. The mechanistic physico–chemical properties of hydrogels can be enhanced by the robust structure of GO/rGO nanoparticles, which also evolve antibacterial activity [19]. Additionally, GO/rGO have outstanding photothermal characteristics in the near-infrared (NIR) range, allowing them to absorb light radiation and transform their energy into heat that can be used to kill bacterial infections [20]. From this point forward, the various publications on the biomedical uses of GO and rGO motivate us to carefully examine their unusual biological features in order to create new therapeutic treatment approaches for wound healing. 

From the literature review, no study has been carried out on GO/rGO-based aloe vera nanocomposite gels used as wound healing formulations. Hence, the present study illustrates the synthesis and characterization of GO and rGO, followed by in vitro and in vivo analysis to determine angiogenesis and wound healing ability of GO/rGO based hydrogel/aloe vera nanocomposite gels.

## 2. Results and Discussion

### 2.1. Fourier–Transform Infra–Red Spectroscopy (FT–IR) Study

**Figure 1:**
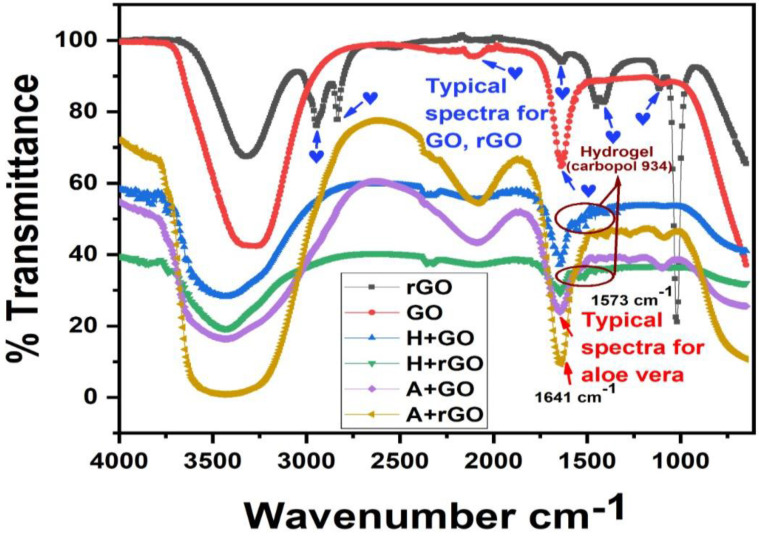
The FT–IR peaks of GO and rGO-based composite gels are shown. GO, which is formed as an oxidized product of graphite, contains an abundance of oxygen containing functional groups, which can be inferred from the strong intensity bands corresponding to the O–H, CH_2_, CH_3_, carboxylic, epoxy, and alkoxy C–O–C bond stretching. After the GO reduction process, the decrease in intensities or absence of oxygen-derived functional groups indicates the absence of oxygen-based functional groups.

The presence of characteristic functional groups of the synthesized GO and rGO and the composite gels were investigated from the vibrational FT–IR spectrum within the wavelength range of 4000 cm^−1^ to 400 cm^−1^ (Figure 1). GO, which is formed as an oxidized product of graphite, contains abundant oxygen containing functional groups, which can be inferred from the strong intensity bands at 3324.6 cm^−1^ corresponding to the O–H bond stretching; 2946.5 cm^−1^ and 2834.7 cm^−1^ indicating the presence of CH_2_ and CH_3_ bond stretching, respectively. The peak at 1638.2 cm^−1^ presents the C=O bond stretching, and 1449.9 cm^−1^, 1412 cm^−1^, and 1114.5 cm^−1^ all correspond to the carboxylic, epoxy, and alkoxy C–O–C bond stretching, respectively [21,22]. This strongly confirms the presence of abundant oxygen-containing functional groups, which in turn corresponds to the presence of an oxidized form of graphite. However, the characteristic peaks of rGO peaked at 3885.6 cm^−1^ corresponding to the hydroxyl group (O–H) group; CH_2_ bond stretching was presented at 2184.2 cm^−1^; 2104.1 cm^−1^ corresponded to CH_3_ bond stretching; and C=O bond stretching at 1638.2 cm^−1^. It can be inferred from the low intensity of the O–H bond and C=O bond stretching peaks that there is a significant decrease in the oxygen containing groups in the graphene planar sheets, confirming the reduction of GO into rGO. 

Multiple peaks connected to oxygen-derived species can be seen in the GO spectrum. Additionally, the sudden decrease in intensities or absence of them in the oxygen-derived functional groups represents the precise reduction reaction of rGO from its precursor material of GO. However, the characteristic peaks for the aloe vera are seen near the wave-length range of 3400 cm^−1^ and 1641 cm^−1^, which corresponds to the hydroxyl group stretching which is observed in uronic acid and mannose and the carboxylate group (–COO group) stretching which is present in the functional compounds of the aloe vera [23,24]. Similarly, carbopol–934-based hydrogels correspond to the peaks at a wavelength range of 1527 cm^−1^ and 1452 cm^−1^, presenting the C=O bond stretching and carboxylic bond, respectively [25].

### 2.2. Raman Spectroscopy Analysis

**Figure 2:**
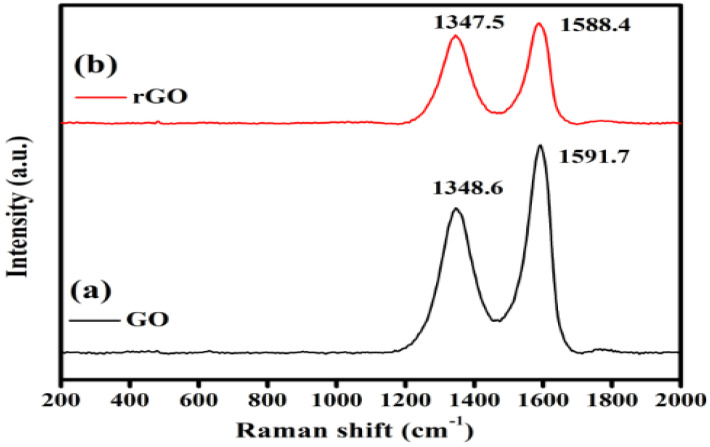
Raman spectra of GO and rGO. The GO and reduction of GO can be identified in Raman spectra by the changes in relative intensity of the two major peaks of the D and G band.

Raman spectroscopy has been widely used to characterize crystal structure and disorder in graphene-based materials. Figure 2 displays the Raman spectra of graphene oxide (GO) and reduced graphene oxide (rGO) in the synthesized samples. The GO and reduction of GO can be identified in Raman spectra by the changes in relative intensity of the two major peaks, which is the D and G band, respectively. The characteristic peak positions of the D and G bands were denoted as 1348.6 cm^−1^ and 1591.7 cm^−1^, respectively. This corresponds to the sp^2^/sp^3^ carbon hybridizations, and the shift in the G peak to 1591.7 cm^−1^ corresponds to the graphitic oxygenation, thus confirming the presence and synthesis of GO. Raman spectra also showed characteristic peaks of rGO at 1347.5 cm^−1^ and 1588.4 cm^−1^ belonging to the D and G band, respectively [26,27]. However, the appeared Raman peaks of rGO do not show a significant change from those of GO, except that the intensity ratio around ID/IG has declined with the reduction. The ID/IG intensity ratio for rGO is larger than that for GO. The slightly larger ID/IG value of rGO (0.848) compared to that of GO (0.847) corresponds to the larger defects and disorder in the carbon materials. This increase in the sp^2^ domain and the higher intensity in the D band in the rGO peak confirm the reduction process, thus validating the removal of oxygen moieties from the GO. The results observed here agree with the previous findings on the GO and rGO [28].

### 2.3. Scanning Electron Microscopy (SEM) and Transmission Electron Microscopy (TEM) Analysis

**Figure 3:**
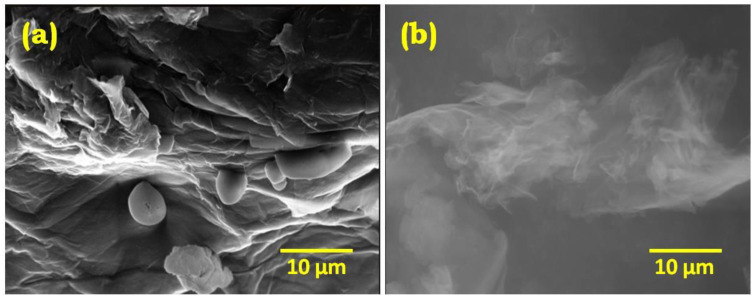
SEM images of (**a**) GO and (**b**) rGO. The contrast difference in the layered and thin transparent layer is observed for GO and rGO in the SEM micrograph, confirming the process of reduction of rGO from GO.

**Figure 4:**
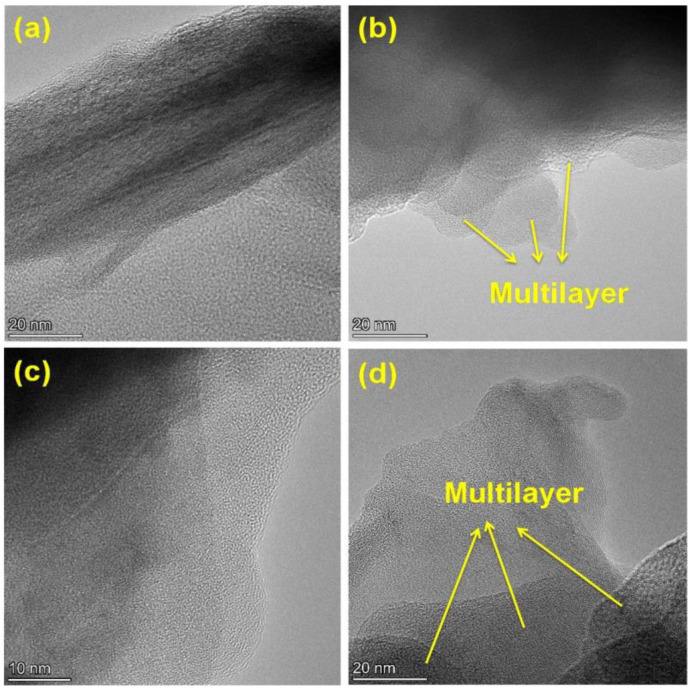
TEM images of GO (**a**,**b**) and rGO (**c**,**d**). The TEM images of GO and rGO show a layered structure and flat multilayer morphology.

The morphological characteristics of GO and rGO were confirmed using scanning electron microscopy (Figure 3a,b) and transmission electron microscopy (Figure 4a–d). A thin and transparent layered sheet structure was observed from the rGO image [29,30]. However, a compact packing of layered and wrinkled flake like structures implied the presence of fully oxidized graphene oxide and the successful delamination of graphite powder during the oxidation process by a modified Hummers method.

The TEM image of GO shows a highly corrugated structure. It is clearly observed from the dark shaded regions, which indicate the layered fashion of the graphene oxide layers [31]. Additionally, it can be observed from the TEM image of GO (Figure 4a,b), the slightly folded and curly edges that correspond to the attachment of the oxygen containing functional groups in the edges of the stacked graphene oxide layers. However, the TEM image showed a clean and disordered, single, multilayered structure [32]. Due to the reduction process, there is a disintegration in the orderly stacked layers of graphene oxide, resulting in a flat morphology [33]. Additionally, the reduction caused the absence of many oxygen-containing functional groups that can be observed from the TEM image of rGO (Figure 4c,d) which has almost smooth edged lattice fringes and decreased wrinkles [34].

### 2.4. Physico–Chemical Parameters of Composite Gels

All the gel formulations (aloe vera, hydrogel, aloe vera + GO, aloe vera + rGO, hydrogel + GO and hydrogel + rGO) achieved a homogeneous and almost transparent nature which helped in monitoring the wound area upon their application [35,36,37]. The simple mixing of the GO and rGO in the hydrogel and aloe vera gel imparted the added advantage of processing the formulation at room temperature without using any additional cross-linking agents, which might hinder the purity of the formulation. Henceforth, the formulations reported here are safe for clinical applications.

### 2.5. Spreadability

**Figure 5:**
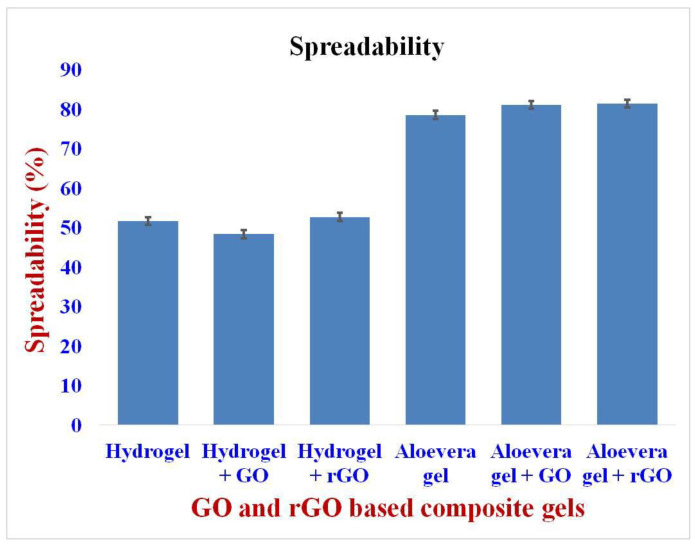
Spreadability of GO and rGO-based composite gels. Aloe vera gels are more spreadable than hydrogels.

The spreadable nature of the synthesized gels was studied, and the gels reported here showed a high spreadability nature. The spreadability of the gels is an important factor in determining their application to the wound surface. Poor spreadability indicates that the drug compound, GO and rGO, is applied to the wound surface unevenly. The hydrogel-based composite formulation showed a spreadability percentage of around 51.62% (Figure 5), whereas the aloe vera-based formulations showed a spreadability of around 78% to 80% [38,39]. The results showed that the aloe vera gels are more spreadable when compared to the hydrogel, resulting in a uniform application over the wound surface [39,40]. The high spreadability of aloe vera gel is due to its high-water content and its naturally homogeneous character. Additionally, as a derivative of graphene, GO and rGO possess mechanical and tribological properties, thus enhancing the spreadability of the composite gels to some extent. This property is due to the presence of sp^2^ hybridized carbon atoms, and the various functional groups present between and around the graphene layers [41,42].

### 2.6. Solubility

**Figure 6:**
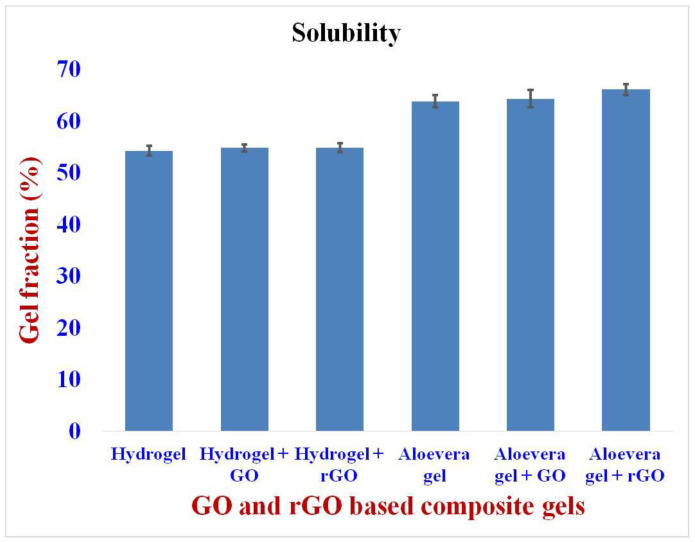
Solubility of GO and rGO-based composite gels.

The solubility of the formulations was carried out by immersing the composite gels in de-ionized water. The carbopol hydrogel showed a lower solubility of 54% compared to that of the aloe vera gel, which showed an insoluble gel fraction of about 60% (Figure 6). Hence, it is clear that the aloe vera-based composite gels are more readily soluble than the hydrogels [40]. Additionally, from the previous literature, it is proven that the GO and rGO are readily soluble in polar and non-polar solvents, which depend upon the presence and type of functional groups in the graphene layers [43]. Henceforth, it is one of the major reasons for the enhancement in the solubility range of the composite gels used here to a greater extent.

### 2.7. Swellability

**Figure 7:**
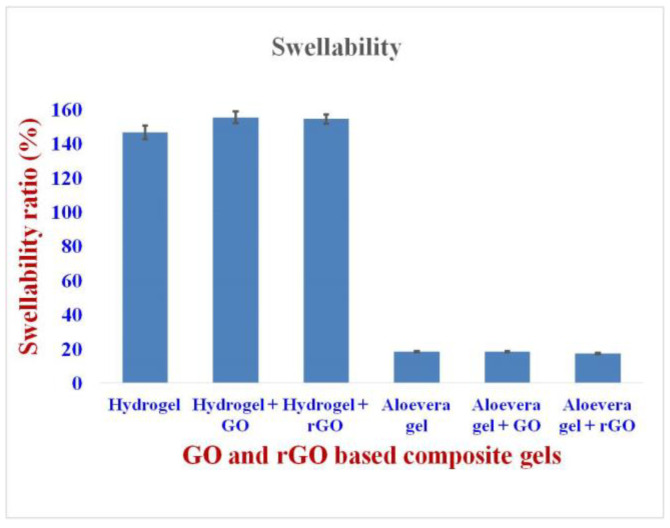
Swellability of GO and rGO-based composite gels. The hydrogel showed a swelling index of around 150%, whereas the aloe vera gel showed a very low swelling index of about 18%.

The swelling ability of the hydrogel formulations was high when compared to the aloe vera gel formulations. The hydrogel showed a swelling index of around 150% whereas the aloe vera gel showed a very low swelling index of about 18% (Figure 7). This is attributed to the highly porous nature of the hydrogel, which in turn possessing high surface area for absorbing the wound exudates [44]. It also showed the inter-relationship between the type of polymer present, water content, and the ionic strength [45]. The high swelling nature of the hydrogel clearly exhibits the benefit of absorbing exudate from the wound surface and its water holding capacity. In comparison, aloe vera gel demonstrated poor absorbing capacity, resulting in a moist environment throughout the wound healing process.

### 2.8. Antioxidant Activity

**Figure 8:**
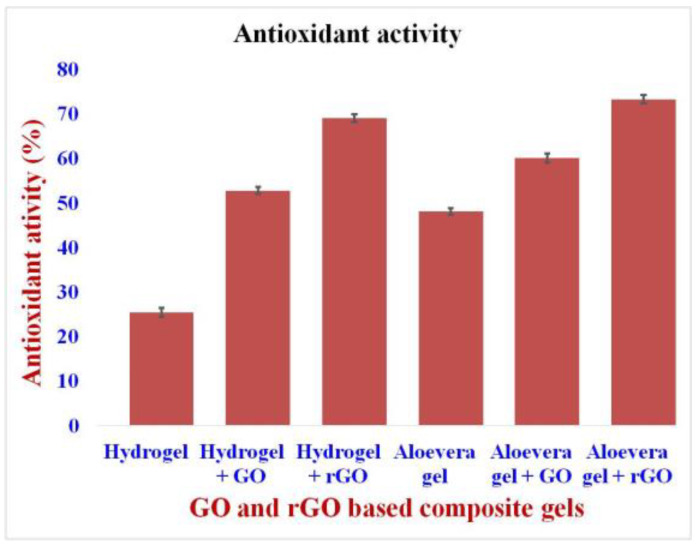
Antioxidant activity of GO and rGO-based Composite Gels. The antioxidant activity of the formulations was carried out using a DPPH assay. rGO-based hydrogel and aloe vera gel (hydrogel + rGO and aloe vera + rGO) showed a high percentage of antioxidant activity.

The antioxidant activity of the formulations was carried out using a DPPH assay. In this assay, the rGO-based hydrogel and aloe vera gel (hydrogel + rGO and aloe vera + rGO) showed a high percentage of antioxidant activity of about 68.9% and 73.2%, respectively (Figure 8). It is due to the reduced activity of rGO. However, GO-based gels (hydrogel + GO and aloe vera + GO) showed a lesser percent of antioxidant activity when compared to that of rGO-based gels, at about 52% and 60%, respectively [46,47]. The pure hydrogel showed the least antioxidant activity of about 25% and the pure aloe vera gel showed an antioxidant activity of about 48%. It is well known from previous literature that the aloe vera gel has natural antioxidant properties, which help in the faster healing of infectious wounds [44,48]. Hence, from the results, it is clear that the rGO-based aloe vera formulation showed a better result for antioxidant activity in wound healing. It is a well-known fact from the previous literature that graphene oxide and reduced graphene oxide show exceptional free radical scavenging activity. This is due to the structure of the layered GO/rGO and the presence of sp^2^ carbon centers within them, which form an adduct with the free radicals, electron transfer, and the donation of hydrogen from their many functional groups, thus effectively inhibiting free radical generation [49].

### 2.9. Hemocompatibility Assay

**Figure 9:**
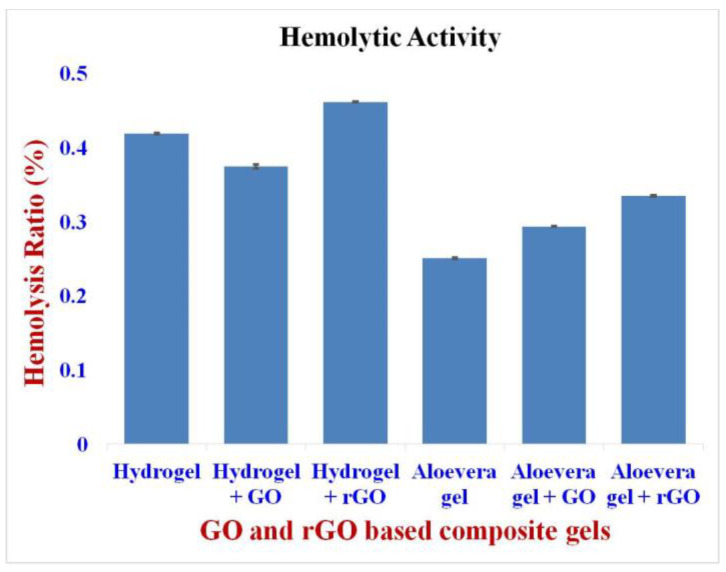
Hemolytic activity of GO and rGO-based composite gels. The hemolytic activity of the GO and rGO-based formulations showed an accepted hemolytic value of less than 2%.

The hemolytic activity of the GO and rGO-based formulations showed an accepted hemolytic value of less than 2%. The hydrogel-based formulations showed the following hemolytic activity: Hydrogel–0.41%; hydrogel + GO–0.37%; and hydrogel + rGO–0.46%, respectively. Similarly, the aloe vera-based formulations showed a hemolytic activities of: aloe vera–0.25%; aloe vera + GO–0.29%; and aloe vera + rGO–0.33%, respectively [50] (Figure 9). From these results, it is clear that the GO and rGO are non-hemolytic, and their conjugation with hydrogel and aloe vera gel has good blood compatibility. It also shows that aloe vera is much more compatible when compared to hydrogel.

### 2.10. Antibacterial Assay

**Figure 10:**
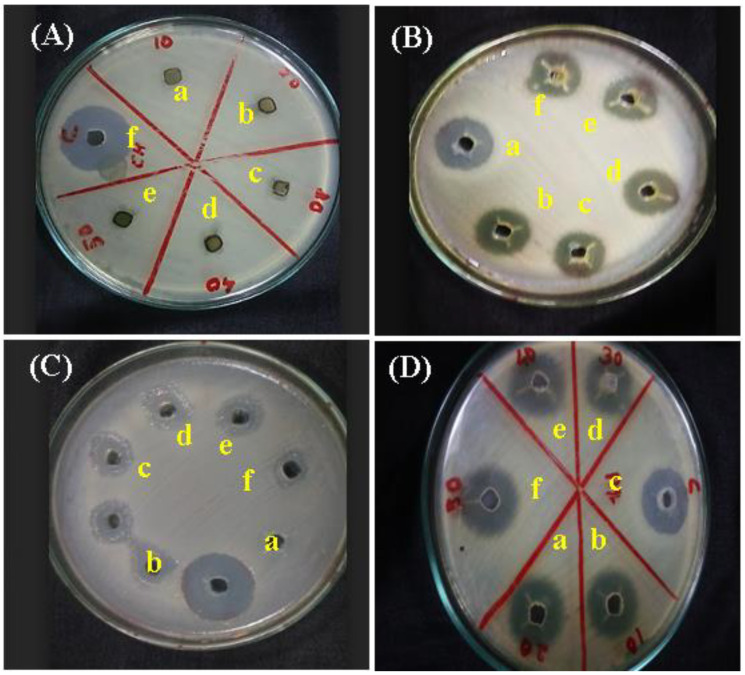
Agar Well diffusion antibiotic sensitivity assay with (a) hydrogel; (b) hydrogel + GO; (c) hydrogel + rGO; (d) aloe vera gel; (e) aloe vera gel + GO; (f) aloe vera gel + rGO against (**A**) *Pseudomonas aeruginosa*, (**B**) *Bacillus subtilis*, (**C**) *Staphylococcus aureus* and (**D**) *E. coli*.

After one day of incubation, the plates were observed to measure the inhibition zones produced by the respective organisms, as shown in Figure 10. It was clear that the activity produced by rGO-based formulations was higher compared to GO-based formulations. The combined effects of rGO and hydrogel/aloe vera gel were enhanced when compared to GO [20,51]. Also, aloe vera-based formulations had better antibacterial activity compared to hydrogel-based formulations. Hence, it is clear that rGO conjugated in aloe vera gel showed a greater activity followed by rGO in hydrogel which aids in the wound healing activity. Table 1 shows the zone of inhibition assay using a diffusion antibiotic sensitive assay. The previous literature shows that the graphene family is a potent anti-bacterial compound that inhibits the growth of bacterial species by reactive oxygen species dependent oxidative stress mechanisms and by suppressing the bacterial film by graphene layers [52]. Thus, the obvious results of our study show that the incorporation of GO and rGO to the gel considerably inhibited the formation of bacterial infection, hastening the wound healing process.

### 2.11. In Vitro Cytotoxicity Assay

**Figure 11:**
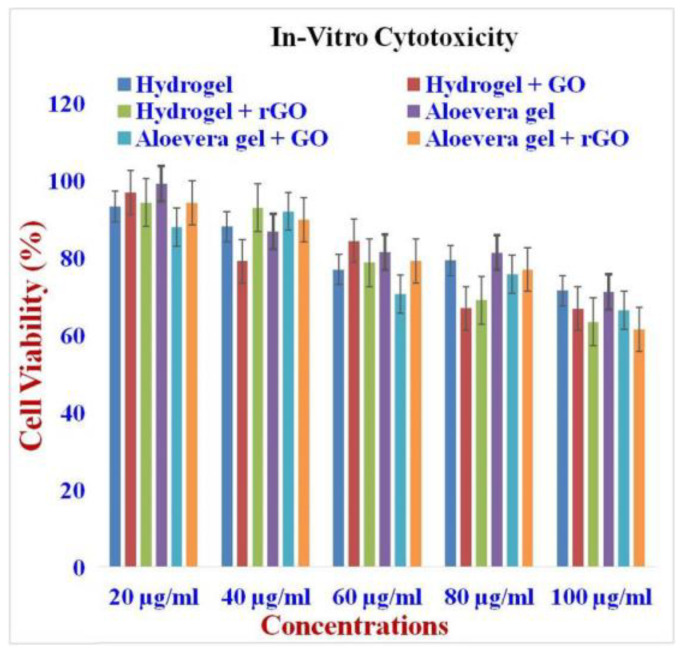
In vitro Cytotoxicity of GO and rGO-based composite gels. The cytotoxicity of the GO and rGO-based formulations showed no toxic effect on the fibroblast cell line.

**Figure 12:**
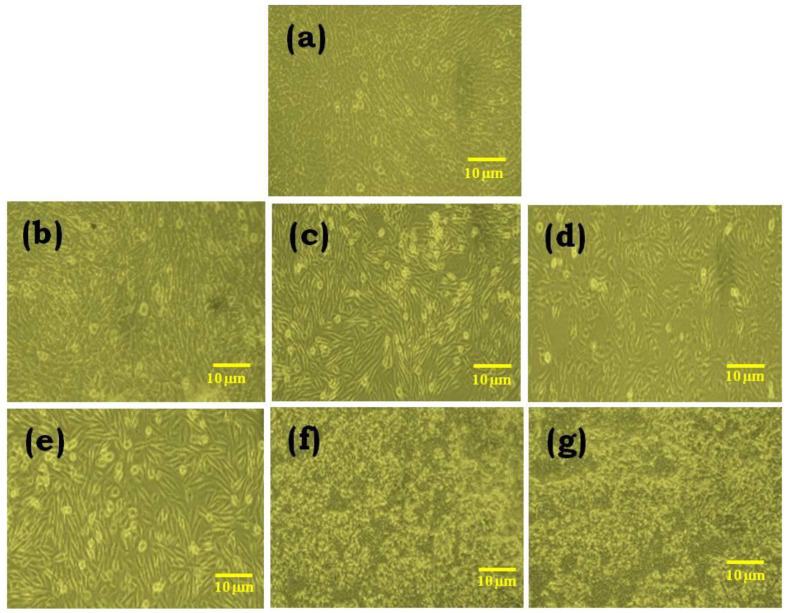
Cytotoxicity Assay of GO and rGO-based composite gels on NIH 3T3 cell line. (**a**) control; (**b**) hydrogel; (**c**) hydrogel + GO; (**d**) hydrogel + rGO; (**e**) aloe vera gel; (**f**) aloe vera gel + GO; (**g**) aloe vera gel + rGO.

Cell viability of the GO and rGO-based gel formulations on NIH 3T3 fibroblast cells was investigated by MTT assay, and its results are presented in Figure 11. The cytotoxicity of the GO and rGO-based formulations showed no toxic effect on the fibroblast cell line. In particular, the aloe vera-based formulations showed the least cytotoxicity when compared to that of hydrogel-based formulations [53]. This characteristic result is due to the natural origin of aloe vera, which impacts the wound healing process in a positive manner. Cell proliferation and morphology of the cells were DAPI stained, which showed the living and fixed cells qualitatively (Figure 12). Based on these cytotoxicity assays, it is shown that the best GO and rGO-based hydrogel and aloe vera gel formulations are biocompatible and suitable for clinical applications, particularly wound healing [54]. On average, 80% of the fibroblast cells were metabolically active at higher concentrations, whereas at lower concentrations, 90% of the cells were actively present, which indicated the modest cytotoxic effects of the GO and rGO [55].

### 2.12. Wound Scratch Assay

**Figure 13:**
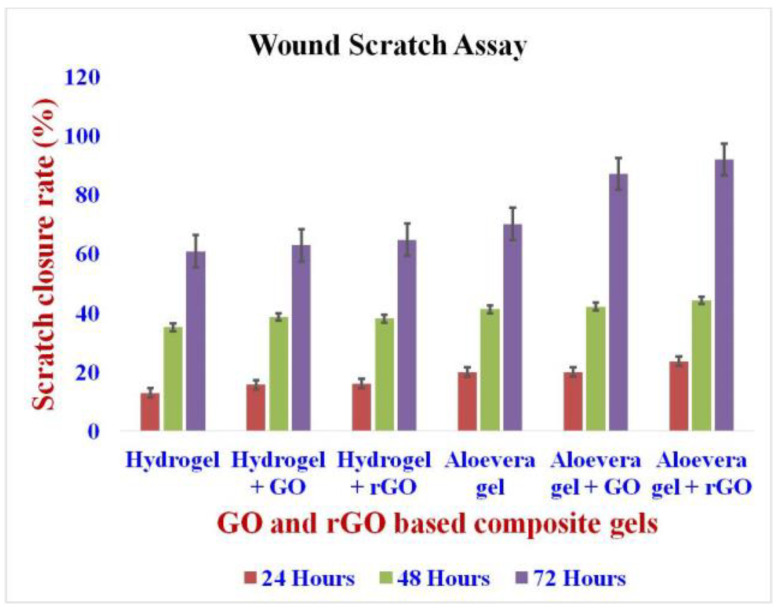
Wound scratch assay of GO and rGO-based composite gels. The study revealed a high level of cell migration, thus revealing significant wound scratch closure.

**Figure 14:**
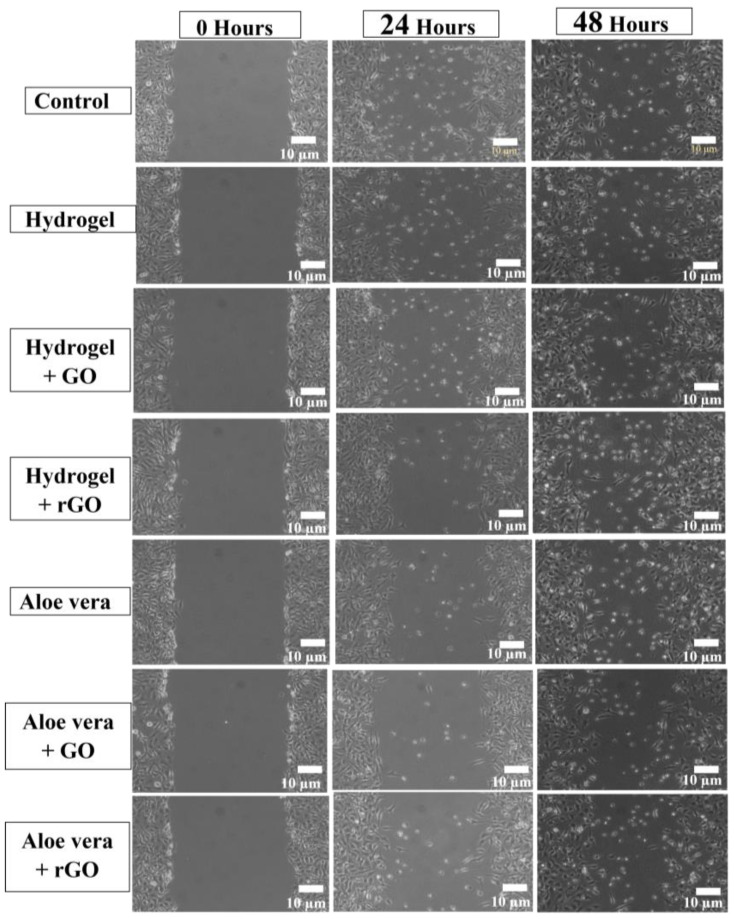
Wound scratch assay of GO and rGO-based composite gels on NIH 3T3 cell line. The wound scratch assay performed on the NIH 3T3 fibroblast cell reveals the wound healing capacity of the rGO-based formulations, which were better when compared to the GO-based formulations and the pure hydrogel/aloe vera gel.

The wound scratch assay performed on the NIH 3T3 fibroblast cell reveals the wound healing capacity of the rGO-based formulations, which were better when compared to the GO-based formulations and the pure hydrogel/aloe vera gel (Figure 13 and Figure 14) [56]. In comparison, aloe vera gel-based formulations also showed a better wound closure rate when compared to hydrogel-based formulations. Hence, the combination of rGO and aloe vera gel had the greatest wound healing capacity.

### 2.13. In Vivo Studies: Wound Contraction Rate

**Figure 15:**
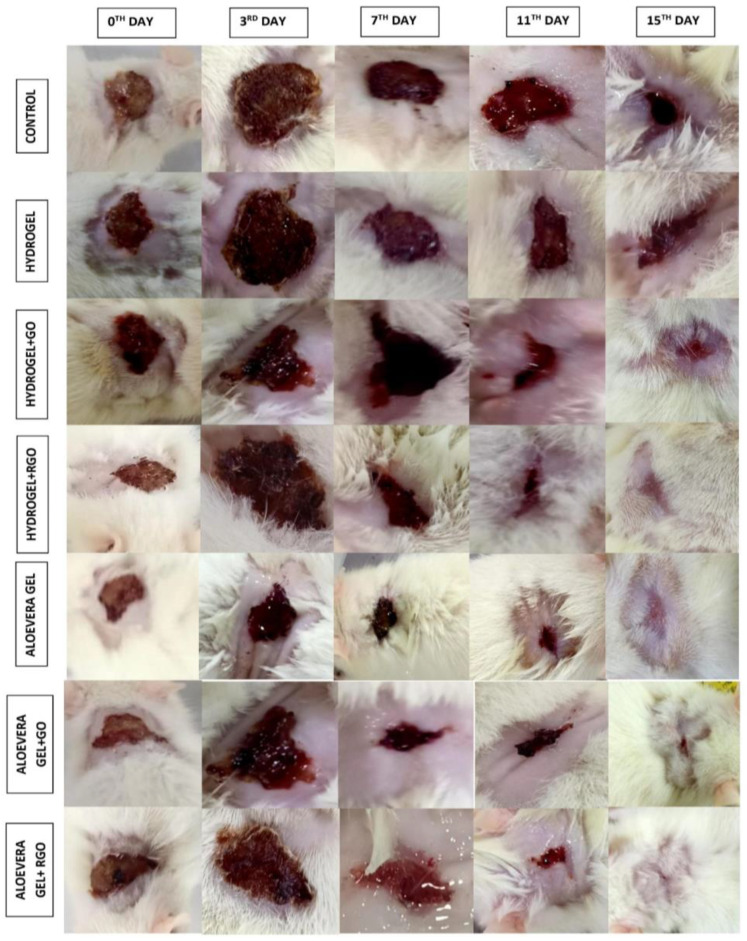
Wound contraction rate of GO and rGO-based composite gels. The measurement of wound contraction is a major standard for the indication of radical wound healing which is expressed as the decrement in the wound diameter from the surgery date (0th, 3rd, 7th, 11th, and 14th days).

**Figure 16:**
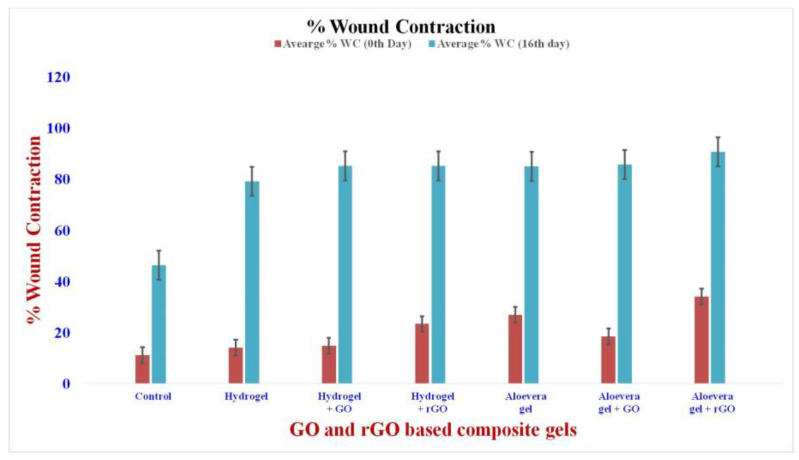
In vivo wound contraction of GO and rGO-based composite gels.

Infections were induced in the open wounds of male Wistar rats by inoculating them with Staphylococcus aureus. The formulations were daily applied over the wound surface for the treatment groups for 14 days (Figure 15). The measurement of wound contraction is a major standard for the indication of radical wound healing [57], which is expressed as the decrement in the wound diameter from the surgery date. All wound contraction was calculated by tracing out the wound surface every 0th, 3rd, 7th, 11th, and 14th days (Figure 16). The wound treated with the composite gels showed a significant decrease in the wound area during the course of treatment. At the end of the treatment period of 14 days, the rGO-based aloe vera gel showed the best wound contraction rate, followed by rGO-based hydrogel formulation. This shows that the rGO best imparts the wound healing property compared to that of GO. Henceforth, its conjugation with hydrogel and aloe vera gel showed almost the same results of wound contraction and hastened the healing property.

### 2.14. Histopathology

**Figure 17:**
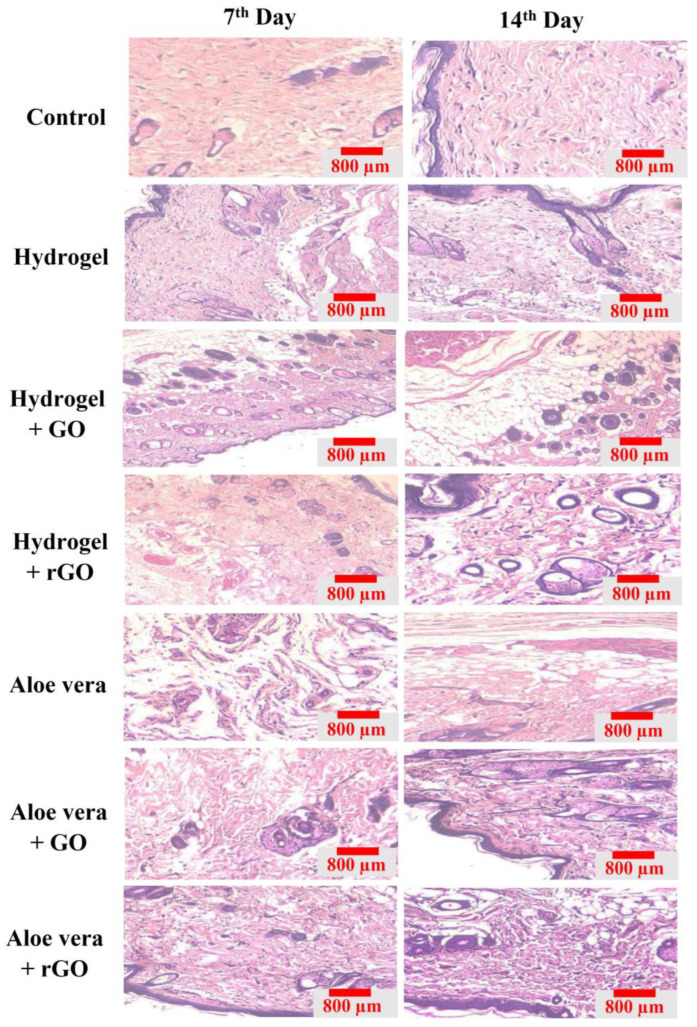
H&E stained granulation tissues of GO and rGO-based composite gels on the 7th and 15th days. Day 7 and 15 tissue samples stained with H&E stain from the treatment groups showed the formation of the epithelial tissue layer, the presence of dense polymorphonuclear cells, and blood vessel formation. Day 15 tissue samples stained with H&E stain from the treatment groups showed the formation of the epithelial tissue layer, the presence of dense polymorphonuclear cells, and blood vessel formation.

**Figure 18:**
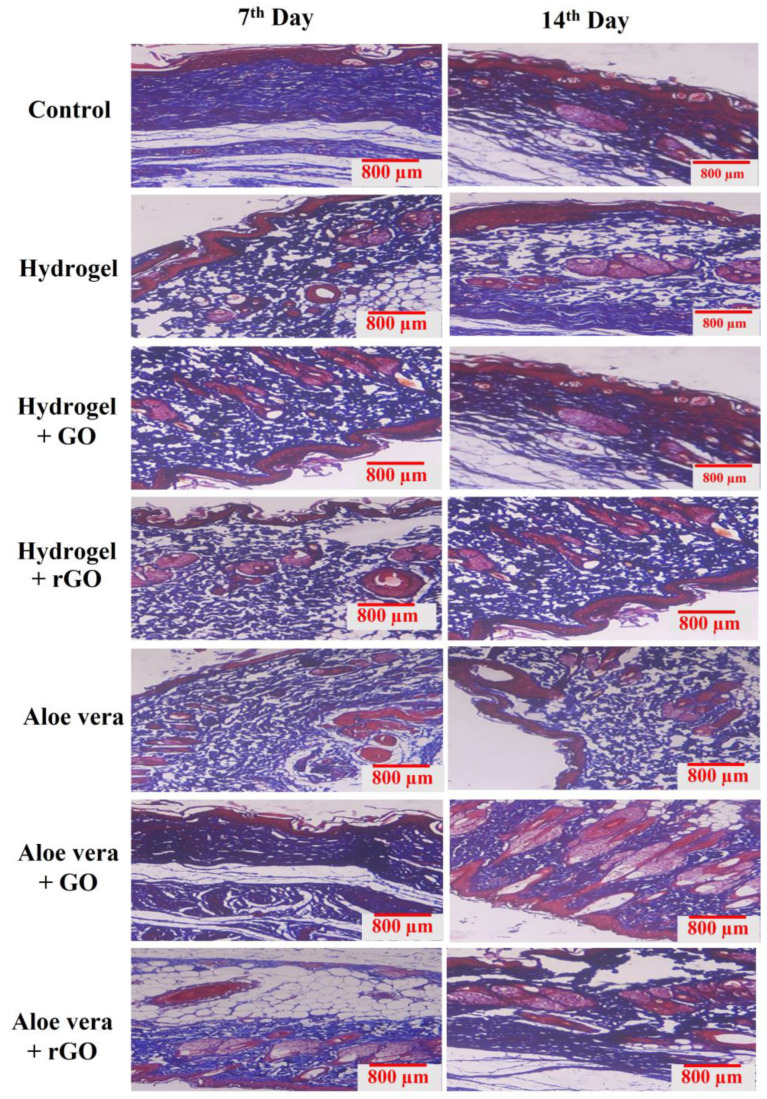
Masons trichome stained granulation tissues of GO and rGO-based composite gels on 7th and 15th days. The increased collagen deposition can be seen in the Masons trichome stained tissues of 14th day when compared to the 7th day.

The granulation tissues collected from the treatment groups on the 16th day were fixed in paraffin wax and used for histology analysis. The sectioned tissues were stained with hematoxylin and eosin (H&E) (Abbey Color, Philadelphia, PA, USA) and Masons trichome stains (San Francisco, CA, USA). Day 15 tissue samples stained with H&E stain from the treatment groups showed the formation of the epithelial tissue layer, the presence of dense polymorphonuclear cells, and blood vessel formation (Figure 17). The treatment groups based on the aloe vera gel especially the rGO + aloe vera group showed a high amount of epithelium regeneration, inflammatory cell infiltration, and the formation of new blood capillaries, which showed an indication of wound the healing process. Similarly, increased collagen deposition can be seen from the masons trichome stained tissues, indicating that treatment incorporation significantly improved collagen synthesis (Figure 18). In particular, the treatment groups containing rGO-based hydrogel and aloe vera gel showed more dense and organized deposition of collagen on the wound beds compared to the control groups and GO-based composite gels. Henceforth, these results demonstrate that aloe vera + rGO and hydrogel + rGO resulted in the best infectious wound healing results, which are induced by epidermis formation and collagen deposition.

### 2.15. Statistical Analysis

**Figure 19:**
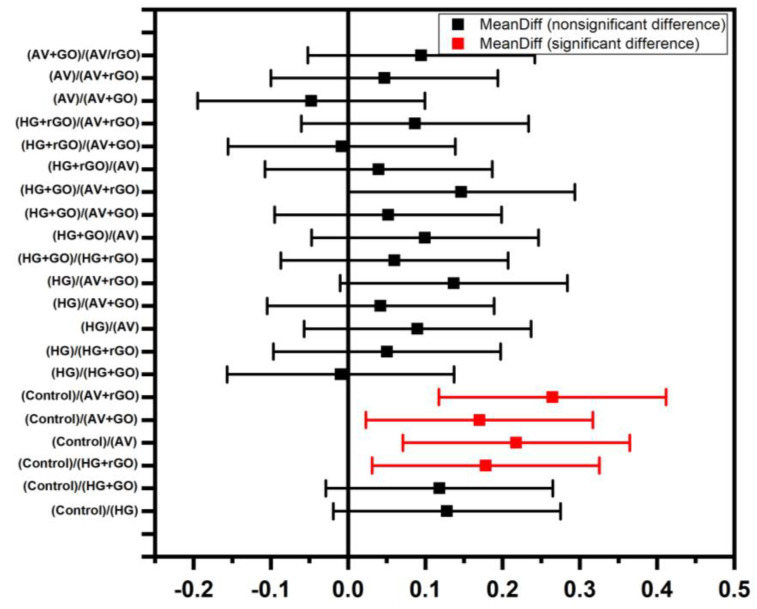
Statistical significance among experimental data using one-way ANNOVA (*p* < 0.5 and *p* < 0.1).

All the reported results were analyzed for their statistical significance, which was studied using one-way ANOVA (*p* < 0.5 and *p* < 0.1). There was a significant healing of wounds as a function of time in all groups, and there (Figure 19) was a significant difference between the groups (*p* < 0.5). From the study, it was evidently proven that wound healing capability was significantly improved in the presence of GO and rGO.

## 3. Conclusions

The current study proposed and validated the application of GO/rGO-based hydrogel and aloe vera gel combination for infectious wound healing. The in vitro cytotoxicity and scratch assay studies using NIH 3T3 fibroblast cells suggest that GO and rGO play an important role in inducing rapid infectious wound healing when conjugated with aloe vera gel. Histological examination depicted a reduction in necrosis, an increase in the production of granulation tissue, and re-epithelialization in the treated Wistar rats using GO/rGO-based hydrogel products. Furthermore, the addition of aloe vera along with GO/rGO, triggered higher levels of collagen formation and maturation, which helps treat infectious wounds. Furthermore, our novel formulation did not only cover the wound surface and absorb the exudate but also promote tissue regeneration and wound recovery. Hence, the proposed rGO-incorporated aloe vera gel delivery system proves to be a safe, cost-effective, natural, biocompatible wound healing therapeutic agent that can be promising in clinical wound care.

## 4. Materials and Methods

### 4.1. Materials

Carbopol–934, hydrazine hydrate, 1,1-diphenyl-2-picrylhydrazyl free radicals (DPPH), Dulbeccos Modified Eagle Media (DMEM), 3-(4,5-dimethylthiazol-2-yl)-2,5-diphenyltetrazolium bromide (MTT), fetal bovine serum (FBS), penicillin, streptomycin—Sigma Aldrich, (St. Louis, MO, USA). Ethanol, glycerol, sulphuric acid (H_2_SO_4_), ortho–phosphoric acid 85% (H_3_PO_4_), Hydrogen Peroxide 30% (H_2_O_2_)—Merck Millipore, (Mumbai, India). Potassium permanganate (KMnO_4_), hydrochloric acid (HCl), glycerol, methyl paraben, D–Sorbitol—SRL Chemical, (Mumbai, India).

### 4.2. Extraction of Aloe Vera Gel

The aloe vera leaves were washed with water to eliminate dirt from the pulp. The aloe vera is cut right at the basal end and is left immersed in water to remove the exudates from the leaves. Following this, the green epidermis was carefully peeled off from the fleshy parenchyma using a knife. The parenchymal flesh is repeatedly washed with water to remove the residual exudates from their surfaces. The flesh is then homogenized in a blender and filtered using polyester cloth material. After that, the aloe vera gel was stored at 4 °C for further use [16,56].

### 4.3. Hydrogel Preparation

0.5 g of Carbopol 934 polymer was dispersed gently into 50 mL of deionized water with constant stirring using a magnetic stirrer at 37 °C to avoid any visible lumps in the dispersion [15]. To the stirring dispersion, an alcoholic solution of glycerol (1:9 ratio of ethanol:glycerol) was added with constant stirring, and a homogeneous dispersion was maintained. To the mixture, a known quantity of permeability enhancers like D–Sorbitol and an anti-microbial peptide (methyl paraben) was added [12,13].

### 4.4. Synthesis of Graphene Oxide (GO) 

GO was created by employing a modified version of Hummers’ technique using graphite powder [57]. H_3_PO_4_ and H_2_SO_4_ were combined in a mixture with a volume ratio of 1:9 (20:180 mL). After stirring the mixture for 15 min, 1.5 g of graphite powder is added. 9.0 g of KMnO_4_ was then continuously stirred with the mixture for about 72 h [58]. To break the reaction, 4 mL of 30% hydrogen peroxide (H_2_O_2_) is combined after 72 h. By adding hydrochloric acid (HCl) and deionized water and subsequent centrifugation (5000 rpm for 15 min) for each washing phase, the mixture was split and cleaned in a centrifuge. After two iterations, the ultimate result was obtained.

### 4.5. Synthesis of Reduced Graphene Oxide (rGO)

A GO dispersion was made by dissolving 1.5 g of GO powder in 500 mL of DI water and stirred continuously for around 30 min. In addition, 0.5 mL of hydrazine hydrate is gently mixed with the dispersion. The mixture was then continuously stirred while being heated to 80 °C in an oil bath. After that, HCl and DI water will be added, and subsequent centrifugation was done to split the mixture phase and cleaned it in a centrifuge. The process was performed twice, and the final product was dried for 24 h at 80 °C in the oven [59].

### 4.6. Characterization 

GO and rGO were analyzed for their physico–chemical parameters. Raman spectroscopy (Renishaw, UK) was used to inspect the quality of GO and rGO at a wavelength of 1000–4000 cm^−1^ [60]. Scanning electron microscopy (SEM, Carl Zeiss, Wetzlar, Germany) and transmission electron microscopy (TEM, Thermo Scientific TALOS F200S G2, Waltham, MA, USA) were employed to determine the surface morphology of the samples. Further, Fourier Transform Infra–Red (FT–IR, PerkinElmer-1600, Waltham, MA, USA) was used to determine the functional groups present in GO and rGO in the wavelength range of 4000–400 cm^−1^ [51,61,62].

### 4.7. Preparation of Composite Wound Healing Gels

0.05% of powdered GO and rGO were blended with the freshly prepared hydrogel and aloe vera gel extracts, respectively [18].

### 4.8. Visual Examination

The prepared GO and rGO conjugated hydrogels and aloe vera gels were examined for their colour, homogeneity, and presence of any lumps by visualization. After the visual observation, the formulations were stored in a transparent plastic container [19].

### 4.9. Spreadability

The spreadability of the hydrogel and aloe vera gel formulations was tested by sandwiching 1 g of the mixture between two horizontal glass slides, followed by the addition of 100 g of standardized weight to the upper slide, where, no further spreading was anticipated, for roughly 5 min [63]. Spread circle diameters were measured in centimeters and used as benchmarks for spreadability [64]. The spreadability of the composite gels was calculated using the following formula.

$$\mathrm{Spreadability}\;=\left[\frac{\mathrm{weight}\;\mathrm{on}\;\mathrm{the}\;\mathrm{upper}\;\mathrm{slide}\times \mathrm{diameter}\;\mathrm{of}\;\mathrm{gel}\;\mathrm{spread}}{\mathrm{time}\;\mathrm{taken}\;\mathrm{in}\;\sec\mathrm{onds}}\right]\times 100$$

### 4.10. Solubility

The hydrogel content of the given hydrogel/aloe vera gel is estimated by measuring the insoluble portion in dried sample after immersing the formulations in de-ionized water for a time period of 16 h at room temperature [65]. The gel fraction of the hydrogel formulation is calculated using the following formula:

$$\mathrm{Gel}\;\mathrm{Fraction}\;\mathrm{of}\;\mathrm{hydrogel}\;=\left[\frac{\mathrm{Initial}\;\mathrm{weight}\;\mathrm{of}\;\mathrm{the}\;\mathrm{gel}}{\mathrm{Dried}\;\mathrm{weight}\;\mathrm{of}\;\mathrm{the}\;\mathrm{gel}}\right]\times 100$$

### 4.11. Swellability

Swellability is an important factor in exuding wounds [66]. To determine the swellability of the hydrogel/aloe vera gel, one gram of each gel was immersed in 5 mL of phosphate buffer (pH—5.5) and left for 30 min, after which the excess buffers were removed. The weights of the hydrogel and aloe vera gel before and after immersion were noted, and the swelling ratio was calculated using the following formula:

$$\mathrm{Swelling}\;\mathrm{Ratio}\;=\left[\frac{\mathrm{Weight}\;\mathrm{of}\;\mathrm{the}\;\mathrm{swollen}\;\mathrm{gel}-\mathrm{Initial}\;\mathrm{weight}\;\mathrm{of}\;\mathrm{the}\;\mathrm{gel}}{\mathrm{Initial}\;\mathrm{weight}\;\mathrm{of}\;\mathrm{the}\;\mathrm{gel}}\right]\times 100$$

### 4.12. Antibacterial Analysis

The antimicrobial activities of GO and rGO-based composite gels were determined by agar well diffusion assay [22,67]. Streptomycin for bacteria (20 μL), such as *Bacillus subtilis*, *Pseudomonas aeruginosa*, *E. coli* and *Staphylococcus aureus*, were used as positive controls. Wells were made in the agar plate and the bacterial inoculums were spread by T-rod and 60 μL of each sample was loaded onto the well. Finally, the Petri plates were left undisturbed for 24 h at 37 °C. The zone of inhibition was measured.

### 4.13. Antioxidant Analysis

The antioxidant activity of GO/rGO hydrogels and aloe vera gels was determined by the competence of aloe vera/hydrogels to scavenge 1,1-diphenyl-2-picrylhydrazyl free radicals (DPPH) [68]. Various concentrations of hydrogel samples (3, 9, 15, and 21 mg) were homogenized using a tissue grinder and mixed with DPPH (100 μM) in a 3 mL ethanol solution, which was stirred in a dark environment for 30 min. The resultant solution was centrifuged, and the wavelength of DPPH in the supernatant was scanned by a UV-vis spectrophotometer. The scavenging ratio of DPPH was calculated using the following formula:

DPPH scavenging % = (AB − AS)/AB × 100%

where AB is the DPPH absorption of blank (DPPH + ethanol); AS refers the DPPH absorption of hydrogel group (DPPH + ethanol + hydrogel).

### 4.14. In Vitro Cell Culture Studies

The National Centre for Cell Sciences (NCCS), located in Pune, India, is where the NIH 3T3 cell line was acquired. Dulbeccos Modified Eagle Media supplemented with 10% (*v*/*v*) heat inactivated FBS, 100 g/mL penicillin, and 100 g/mL streptomycin were used in in vitro analysis. Cells were kept in the logarithmic phase of growth. The cells were kept at 37 °C in an incubator with 5% CO_2_ and 95% humidified air [69].

#### 4.14.1. Cytotoxicity Assay

GO and rGO were used to determine cytotoxic behavior using the MTT (3-(4,5-dimethylthiazol-2-yl)-2,5-diphenyltetrazolium bromide assay) against the NIH 3T3 cell line. Briefly, each cell line was seeded in a 96-well microplate individually (1 × 10^6^ cells/mL), then the plates were incubated at 37 °C for 24 h with 5% CO_2_, and the cells were allowed to develop until 90% confluence [26,70,71]. After the incubation period, the media was changed, and the cells received treatments of GO and rGO at concentrations ranging from 20, 40, 60, 80, and 100 g/mL. After that, the samples were cultured for 24 h. Following a PBS wash (pH = 7.4), 20 L of MTT solution (5 mg/mL) was added to each well of the cells. The cells were then left to stand at 37 °C in the dark.

$$\mathrm{Cell}\;\mathrm{Viability}\;=\left[\frac{\mathrm{Absorbance}\;\mathrm{of}\;\mathrm{treated}\;\mathrm{cells}}{\mathrm{Absorbance}\;\mathrm{of}\;\mathrm{control}\;\mathrm{cells}}\right]\times 100$$

#### 4.14.2. In Vitro Wound Scratch Assay

The previously reported and established protocol was followed for conducting this experiment [26,27]. In 6-well plates (8 × 10^5^ cells/well), NIH 3T3 cells were plated and cultured under ideal growth conditions until 90% confluence was obtained. To simulate a wound, a scratch was produced in the center of the cell monolayer using a P10 pipette tip, and cell debris was removed by washing with new media. For 48 h at 37 °C in cells that were kept untreated, the wound was exposed to 100 g/mL of aloe vera, hydrogel, aloe vera + GO, aloe vera + rGO, hydrogel + GO, and 50 g/mL of commercial medication (Cipladine; positive control). Four digital photos were collected at various points throughout the analysis of the scratch wound closure using an inverted microscope.

### 4.15. In Vivo Wound–Healing Studies

#### 4.15.1. Animals and Experimental Protocol

Male Wistar rats were used to test the efficacy of GO/rGO conjugated aloe vera/hydrogel on wound healing. All the animal experiments were performed with absolute care and following the ethical guidelines laid out by the Biomedical Research Unit and Laboratory Animal Centre of Saveetha Dental College and Hospitals, India, after obtaining ethical clearance (BRULAC/SDCH/SIMATS/IAEC/3−2021/058). Male Wistar rats of about 3–4 months old, weighing about 150–300 g were sheltered in stainless steel lids covered polypropylene cages and acclimatized for 7 days. The animals were maintained in an air circulated environment with the arrangement of standard 12:12 h light:dark cycles [69]. They were fed with commercial rodent pelleted foods and drinking water in standard intervals and wound healing was monitored during 2–16 days. The male rats were randomized into 7 groups, with 2 animals in every group. The experimental groups of animals were treated as follows:

1. Control;
2. Aloe vera gel;
3. Aloe vera gel + Graphene Oxide (GO);
4. Aloe vera gel + Reduced Graphene Oxide (rGO);
5. Hydrogel;
6. Hydrogel + Graphene Oxide (GO);
7. Hydrogel + reduced Graphene Oxide (rGO).

#### 4.15.2. Anesthesia and Wound Creation in Rats

The pre-operative analgesia was induced by the intra-peritoneal injection of 10 mg/kg of Xylazene and 25 mg/kg of Ketamine. The dorsal area of the rats was placed facing the dissecting pad, and the backs of the anesthetized rats were shaved, cleaned, and disinfected using 70% ethanol. A full thickness circular excisional wound of 20 mm was created on the dorsal interscapular part. Subsequently, the wound was inoculated with Staphylococcus aureus. Two days post wound infection, the wounds were treated daily with aloe vera gel, hydrogel, 0.05% GO + aloe vera gel, 0.05% GO + hydrogel, 0.05% rGO + aloe vera gel, and 0.05% rGO + hydrogel, respectively. The results were compared to the untreated control group. The open wound was traced along the wound margin using transparent OHB sheets to determine the percentage wound contraction rate on every 0 (surgery day), 4, 8, 12, and 16 days post-surgery. On the 16th day, all the animals were sacrificed, and the respective wounds were excised. The wound tissue was fixed and further subjected to hematoxylin and eosin (H&E) and Masons trichome staining for histological analysis of wound healing [72].

#### 4.15.3. Macroscopic Biophysical Analysis

The measurement of wound surface area was used for the macroscopic assessment of the reduction in original wound size by calculating the percentage wound contraction [73]. The formula used for the calculation of percentage wound contraction is as follows:

$$\%\;\mathrm{Wound}\;\mathrm{contraction}\;\mathrm{rate}\;=\left[\frac{\mathrm{Wound}\;\mathrm{surface}\;\mathrm{area}\;\mathrm{on}\;\mathrm{day}\;0-\mathrm{Wound}\;\mathrm{Surface}\;\mathrm{area}\;\mathrm{on}\;\mathrm{Day}\;x}{\mathrm{Wound}\;\mathrm{surface}\;\mathrm{area}\;\mathrm{on}\;\mathrm{day}\;0}\right]\times 100$$

#### 4.15.4. Histopathological Analysis

The granulation tissues removed on the 16th day were used for the histology analysis. The collected wound tissue from each group was fixed in 10% neutral buffered formalin and dehydrated using graded ethanol [57]. These samples were cleaned in xylene and processed routinely into paraffin wax. Further, 4 µm thick embedded tissues were sectioned using a microtome. These sections were then stained using hematoxylin and eosin (H&E) and Masons trichome staining [63]. The sections were imaged using Olympus IX81 light microscope (Tokyo, Japan) to microscopically assess the extent of angiogenesis, amount of granulation tissue, re-epithelialization, inflammation, and the total collagen content.

### 4.16. Statistical Analysis

All the results are presented as mean ± standard deviation. The test data were analyzed by one-way ANOVA. All the statistical analyses (*p* < 0.5) were considered statistically significant.

## Figures and Tables

**Table 1:** Zone of Inhibition (in mm) for the Agar Well diffusion antibiotic sensitivity assay.

Composite Gels	*Pseudomonas aeruginosa* (A)	*Bacillus subtilis* (B)	*Staphylococcus aureus* (C)	*E. coli* (D)
Hydrogel	−	10.01	12.56	12.43
Hydrogel + GO	3.77	8.46	6.32	11.82
Hydrogel + rGO	4.27	7.92	6.47	12.19
Aloe vera Gel	−	9.81	6.92	10.89
Aloe vera Gel + GO	3.06	9.76	5.93	11.10
Aloe vera Gel + rGO	15.75	8.13	3.82	13.27

## Data Availability

Not applicable.
